# The Domestic Filtration of Water

**Published:** 1895-09-28

**Authors:** 


### DEPARTMENT OF HYGIENE.

### THE DOMESTIC FILTRATION OF WATER.

The purity of drinking water has been a subject of much interest to scientists and the profession from very early times, and with the progress of knowledge the meaning of the term purity has gradually become more definite and exact. Before the sciences of chemistry and bacteriology attained their present development, a water was considered pure when it had no appreciable odour or taste and was limpid. From these properties one has passed insensibly into a definition of purity based on chemical considerations and measured numerically by chemical methods. Such determinations as the hardness of a water in parts per 100,000, or total organic nitrogen, or the amount of chlorides and nitrates being used by chemists to indicate the relative purity of different waters. At the present time, however, both of these definitions of purity have, to a certain extent, been dismissed in favour of the modern interpretation of the term purity as indicating sterility. This beneficial examination of drinking waters has, however, not led to any very definite results, since all natural waters are more or less contaminated with micro-organisms, and owing to the rapidity with which they multiply in waters, the numerical data often given showing the number of colonies produced from the organisms in a cubic centimetre of water are of little value in gauging the purity of any particular supply. Many bacteriologists consider that such attempts at counting the bacteria are valueless, and that far more information as to the purity of a water can be obtained by ascertaining the number of varieties of organisms present instead of their actual amount. This idea is based on the assumption that each separate variety is originated in the water supply under examination from different points of ingress, and that therefore the number of varieties present is a measure of the number of possible polluting sources. The fact that the majority of natural wholesome waters contain not only large numbers of bacteria, but also in many cases of bacteria of several distinct species, has, however, considerably modified the views held by biologists as to what should be considered a pure water. Attempts have recently been made to classify the water bacteria into pathogenic and non-pathogenic kinds, but here again difficulties have arisen which have so shaken the faith of many believers in the new definition of purity that they have returned to the chemist for his opinion as to the wholesomeness or otherwise of suspicious waters. This collapse one might almost say of the bacteriological definition of purity has been brought about partly by the erroneous assumption of the earlier bacteriologists that the classification of non-pathogenic and pathogenic organisms coincided with their physical property of liquefying or not liquefying gelatine, and partly owing to the fact that many of the pathogenic organisms present in water either lose their toxicity or remain inert in the presence of non pathogenic varieties. Added to these considerations is the difficulty attending the isolation and identification of pathogenic species in large volumes of water containing non-pathogenic varieties. Notwithstanding these drawbacks the purity of a water at the present day must be judged by its freedom from micro-organisms, and the criteria adopted by hygienists and sanitary authorities should be based upon such considerations. As an ideal pure water is therefore one which is absolutely sterile, it is necessary to consider how such sterility can be readily and safely acquired. At present the householder must rely on his own exertions for effecting this object, since attempts at bacteriological purification made by the larger towns and water supply companies have been shown over and over again to be useless in cases of epidemics, and can only be regarded as precautionary measures which should be rendered as efficient as a first line of defence can be made. The purity of a domestic supply can be obtained by two different methods, viz, either by sterilising the water by heat or by using some form of gieser which shall retard the passage of all the micro-organisms present. The apparatus for sterilising by heat, or "stérilisateurs," as they are called on the Continent, are rapidly becoming more generally known, and the better forms are admirably adapted for the supply of sterile water to hospitals and public institutions, whilst country houses seem also to be indicated. It is beyond the scope of the present article to describe any of these water sterilisers. In this country the form known as "Equifex," introduced from Paris by Messrs. Gerieste, Herscher, and Co., is perhaps the most generally adopted. In dealing with the alternative method we have, however, to insist on the absolute necessity for householders to be on their guard against relying on the efficiency of the majority of household filters when tested from the bacteriological point of view. This is more important at the present time when the summer weather brings with it epidemic outbreaks of typhoid and cholera, and in taking the summer holiday the head of a family cannot be too careful in his inquiries as to the source and purity of the water supply which is to be used during his family's absence from home. Dr. Sims Woodhead and Cartwright Wood a few months ago gave an admirable review of the merits and demerits of the various filters which find favour in this country, and from their systematic examination of these filters have come to the important conclusion that only those of the candle type, of which the Berkefeld and Pasteur Chamberland filters are the best known, fulfil modern conditions of efficiency. Unfortunately hitherto these filters have only been of practical service when adapted to a high pressure of water, since their rate of filtration is so extremely slow under ordinary conditions as to practically negative their use. The rival claims of these two forms of candle filters have been critically discussed by Dr. H. H. Johnston, who in a thesis for the degree of Doctor of Science of Edinburgh University last year described a series of experiments to decide this point. His general conclusions are worth quoting: "The Pasteur Chamberland filter is the best and the only one on which reliance can be placed for permanently sterilising water. Its use is therefore recommended for sterilising drinking water, water used for surgical dressings, and wherever sterilised water is required for any particular purpose." In France for many years past this form of filter has been used in the French army, and General Zuremden in his detailed report draws attention to the fact that a decrease in the death-rate of the French army from epidemic disease can be directly traced to the adoption of this method for purifying the drinking supply. Dr. Vallen, in an examination of the typhoid epidemic which occurred in Paris in 1894, also showed that this epidemic was confined to those barracks which were not supplied with Pasteur filters, while those which had these appliances, even with exceptionally bad water, remained unattacked. Within the last few weeks the authorities at Darjeeling in India have decided upon filtering the whole of the water supply of that town upon this plan, and this installation will be one of the first in which the Pasteur Chamberland system has been adopted on a large scale. The drawback which, we have already pointed out, existed in the use of these filters for household purposes, has also been recently removed, as the proprietors have now placed on the market a household filter which works without a high pressure or head of water. In this domestic form, which we have had an opportunity of examining, a battery of three candles is employed, and these are connected by a special indiarubber communicating tube with a syphon tube to the receptacle below. The three candles being immersed in a vessel charged with the unfiltered water commence to act slowly, and the filtered and sterile water syphons over at the rate of one to two pints per hour. A constant supply of pure drinking water can thus be readily obtained in any house without any greater trouble than that of re-charging the reservoir from time to time. From our examination of this new model we are of the opinion that it fulfils the conditions laid down by the bacteriologists, and is at the present time practically the only form of domestic filter which the profession can recommend to the public.

